# Health and Access to Health Services for People with Disability in Australia: Data and Data Gaps

**DOI:** 10.3390/ijerph182111705

**Published:** 2021-11-08

**Authors:** Nicola Fortune, Rosamond H. Madden, Shane Clifton

**Affiliations:** 1 Centre for Disability Research and Policy, The University of Sydney, Susan Wakil Health Building, Western Ave., Camperdown, NSW 2050, Australia; ros.madden@sydney.edu.au (R.H.M.); drshaneclifton@gmail.com (S.C.); 2 Centre of Research Excellence in Disability and Health, University of Melbourne, 207 Bouverie Str., Carlton, VIC 3053, Australia

**Keywords:** health services, disability, data gaps, disability identification, International Classification of Functioning, Disability and Health (ICF), Convention on the Rights of Persons with Disabilities (CRPD), health statistics, disability statistics, inequalities

## Abstract

The right of people with disability to enjoyment of the highest attainable standard of health without discrimination on the basis of disability is enshrined in the United Nations Convention on the Rights of Persons with Disabilities (CRPD). Among its obligations as a signatory to the CRPD, Australia is required to collect appropriate information, including statistical and research data, to inform development and implementation of policies to give effect to the Convention. In this commentary, we first describe how the International Classification of Functioning, Disability and Health (ICF) conceptual model of disability can be operationalised in statistical data collections, with a focus on how this is achieved in key Australian data sources such that people with disability can be identified as a population group. We then review existing statistical data on health and health service use for people with disability in Australia, highlighting data gaps and limitations. Finally, we outline priorities and considerations for improving data on health and access to health services for people with disability. As well as conceptual, practical, and ethical considerations, a key principle that must guide future disability data development is that people with disability and their representative organisations must be involved and participate fully in the development of disability data and statistics, and in their use.

## 1. Introduction

People with disability have the right to enjoy the highest attainable standard of health without discrimination on the basis of disability, as affirmed in Article 25 of the United Nations Convention on the Rights of Persons with Disabilities (CRPD) [1]. This right is not currently being realised. In Australia, as in other countries across the globe, people with disability experience poorer health outcomes than people without disability [2,3,4,5,6]. At population level, disability-related health disparities are caused in large part by avoidable disadvantage, and not primarily by underlying impairment [7]. As such, governments have a duty to act to reduce these health disparities.

Population statistical data have a crucial role to play in identifying and understanding disability-related inequalities, informing more effective policy interventions, evaluating interventions, and holding key actors to account. Numerous international and Australian reports have highlighted the need for population-level statistical data that can be disaggregated by disability, and the importance of using such data for monitoring and regularly reporting on disability-related inequalities [6,8,9,10]. In Australia, the current policy context has generated a high level of interest in disability data. Key features of this context include the National Disability Insurance Scheme (which reached full rollout in July 2020), the ongoing Royal Commission into Violence, Abuse, Neglect and Exploitation of People with Disability, and the development of a new national disability strategy to guide Australian disability policy for the coming decade.

In the context of health systems and health data, disability has traditionally been treated as an outcome [11]. This is powerfully illustrated by the Global Burden of Disease study: an international epidemiological study that produces estimates of the fatal and non-fatal ‘burden’ attributable to specific diseases and risk factors using the Disability Adjusted Life Year (DALY) metric [12,13]. Considering health inequalities experienced by people with disability requires a wholly different perspective. It means viewing disability as akin to a demographic factor by which health data may be disaggregated [11]. However, the concept of disability is inextricably linked to and defined with reference to health. Thus, use of statistical data to investigate disability-related health inequalities presents complexities that require interrogation.

Statistical classifications provide standard structures for collecting, organising and analysing data [14]. The use of standard statistical classifications as a basis for developing data collections promotes data consistency, so that data from different sources may be compared and potentially used together to generate new information and boost the value of existing data resources. The World Health Organization’s (WHO) Family of International Classifications is a suite of classifications designed to provide internationally consistent information on different aspects of health and the healthcare system [15]. The three core members of the WHO-FIC are the International Classification of Diseases (ICD), the International Classification of Functioning, Disability and Health (ICF), and the International Classification of Health Interventions (ICHI) (currently in development). The ICF has been broadly adopted as the international standard framework and classification for organising and documenting information about functioning and disability [16], and has been used in Australia to underpin the development of disability-related data items in population surveys and administrative data collections [17].

In this paper, we describe how people with disability are identified in key Australian data sources, review available statistical data on health and health service use for people with disability, and outline priorities and considerations to guide efforts to fill data gaps and improve the evidence base.

## 2. Methods

To inform this commentary, we searched for publications relevant to disability data on the websites of Australia’s two main national statistics institutions—the Australian Bureau of Statistics and the Australian Institute of Health and Welfare. Reports and technical documents available from these websites provide information about data relevant to disability available in key statistical data sources, including detailed descriptions of how disability is operationally defined.

We also referred to an Audit of Disability Research in Australia, first conducted in 2014 and updated in 2017 [18]. Finally, we conducted a simple search on ‘national disability statistics in Australia’. Our aim was to identify sources of statistical data in Australia relevant to health and health service use for people with disability, and related research.

## 3. Functioning, Disability and Health

The ICF was endorsed by all WHO Member States in the Fifty-fourth World Health Assembly in 2001. Its development was preceded and informed by decades of worldwide discussion involving a range of service providers, health professionals, and researchers, as well as people with disability and their representative organisations [19,20]. One of the main threads of these discussions was the recognition that the environment plays a significant role in the experience of disability.

The ICF defines the main components of functioning as body functions and structures, activities and participation. It provides classifications of body functions, body structures, activity and participation domains and environmental factors (see Box 1). Environmental factors have a crucial effect (as facilitators or barriers) on people’s functioning and on the creation of disability in many life areas. The ICF represents a biopsychosocial model of disability, combining the medical and social models of disability, thus recognising that disability may require both treatment at the individual level, as well as social and environmental change [19,21]. It shows us a dynamic interaction between a person’s health conditions and contextual factors—relationships that are probabilistic and not deterministic or linearly causal.

Box 1 **Definitions of the components of the ICF, and of functioning and disability.**

**Body functions**—The physiological functions of body systems (including psychological functions). **Body structures**—Anatomical parts of the body, such as organs, limbs and their components. **Impairments**—Problems in body function or structure, such as a significant deviation or loss. **Activity**—The execution of a task or action by an individual. **Participation**—Involvement in a life situation. **Activity limitations**—Difficulties an individual may have in executing activities. **Participation restrictions**—Problems an individual may experience in involvement in life situations. **Environmental factors** make up the physical, social and attitudinal environment in which people live and conduct their lives. These are either barriers to or facilitators of the person’s functioning. **Functioning** is an umbrella term encompassing all body functions, activities and participation. It denotes the positive or neutral aspects of the interaction between a person’s health condition(s) and that individual’s contextual factors (environmental and personal factors). **Disability** is an umbrella term for impairments, activity limitations and participation restrictions. It denotes the negative aspects of the interaction between a person’s health condition(s) and that individual’s contextual factors (environmental and personal factors). Source: WHO, 2001, pp. 3, 8, 10

The ICF was developed in the same era as the CRPD, with the former concerned with classification and data, and the latter with provision of human rights. The ICF and CRPD share common concepts, culture and terms (e.g., environment, barriers, participation), and the subject matter of rights in the CRPD can readily be mapped (or cross-walked) to the ICF domains, demonstrating that the two instruments have broad commonality of content [22,23]. The CRPD requires governments that have ratified the Convention to report on progress with achieving its aims—rights, access and equity—and to collect statistics to help formulate and implement policy and to monitor the Convention (*Articles 31, 33 and 35*). The ICF is designed to enable the collection and comparison of data relating to functioning and disability in many fields and across the world, and it provides a suitable framework to underpin monitoring the implementation of the CRPD [23,24].

Statistical classifications provide the building blocks for sound health information systems [25,26]. Over the past 20 years, the ICF has influenced functioning and disability information, measurement and statistics [24]. It is used in diverse applications, settings and countries. Nevertheless, it is considered that there is a need for “more applications of the full ICF model, including those that incorporate Environmental Factors into applications, such as surveys and measurement instruments” ([24], p.1455). The benefits of doing this are well illustrated by the first National Disability Survey in Ireland in 2006, with survey results showing the substantial impact of environmental factors on people’s functioning [27]. The WHO has called for the wider use of the ICF to increase worldwide disability data quality and consistency [21,28,29].

Australia recognises both the moral and legal framework of the CRPD and the technical framework of the ICF. In 2008, Australia ratified the CRPD, and now makes links to CRPD objectives in major public policies and programs, such as the National Disability Strategy 2010–2020 and National Disability Insurance Scheme [30,31]. Any success of these initiatives should be indicated by relative improvements in participation by people with disability in many life areas and in access to services. The challenge for national statistics in any country is to produce reliable data capable of telling the story of participation, access and equity. Australian statistical organisations such as the ABS and the AIHW adhere to international standards to produce data that enable national and international comparisons, and to capture consistent administrative data nationally. Both organisations, before and since the publication of the ICF, have been advised on statistics and data standards by broadly-based groups that include disability advocacy organisations.

Measuring progress in fulfilling CRPD goals requires data that can be used to compare the experiences of people with disability with those of people without disability, for example, in employment, housing, or access to health services. The ICF does not divide people dichotomously into ‘disabled’ and ‘not disabled’. Therefore, methods are required that enable population data to be disaggregated into groups, according to people’s level of functioning. Such methods should be designed to suit the purpose and context, for example, to define eligibility for services, to estimate disability prevalence, or to monitor progress towards achieving equity.

How can the ICF conception of disability as a dynamic interaction between a person’s health conditions and contextual factors (personal and environmental) be best operationalised in statistical data collections, to enable the production of data for people ‘with’ and ‘without’ disability? The first step is to identify the main questions of interest—those that lend themselves to measurement and quantification—and to articulate the main related purposes and methods to be used [32]. Statistical design and analysis can then draw on the clarity and simplification offered by the classifications of the ICF to help frame the concepts underlying the questions on which evidence is sought and specify relevant data items.

Defining ‘disability status’ is not straightforward, given that:

“The ICF puts the notions of ‘health’ and ‘disability’ in a new light. It acknowledges that every human being can experience some disability. … ICF thus ‘mainstreams’ the experience of disability and recognizes it as a universal human experience. By shifting the focus from cause to impact, it places all health conditions on an equal footing, allowing them to be compared using a common metric—the ruler of health and disability.”

([27], p. 1068)

Designing an operational definition of disability is a complex task that is sometimes implied or simplified rather than fully explicated. A description of the method used in the World Report on Disability illustrates some key points in a general methodology [16]. The method entailed creating a spectrum of difficulty with activities and designing ‘cut points’ in a transparent way, to indicate what is included in the definition of ‘disability’ used for estimation purposes ([27], p. 1066). Similarly, the Model Disability Survey was developed by WHO to collect ‘comprehensive, comparable and relevant disability information’ to monitor the CRPD. It aims to capture ‘how people actually function in multiple domains, given the environmental barriers and facilitators that constitute their real life situation’, by asking about ‘problems in daily life’ ([33], pp.4-5). The data are used to generate a continuum ranging from low to high levels of disability—a metrical scale developed using Item Response Theory. This disability distribution can be partitioned using cut-points to define groups with no, mild, moderate and severe disability for data disaggregation purposes.

Since it was first run in 1982, the Australian Survey of Disability Ageing and Carers (SDAC) has been used to provide prevalence estimates of disability and profiles of people (including older people) who experience difficulties functioning in everyday life. SDAC follows the ICF framework and, broadly speaking, its definitions. Inclusion into the survey is via screening questions focused on a list of long-term health conditions, impairments and activity limitations, including ‘any other health conditions resulting in a restriction’. Survey respondents are then asked an array of questions, including about difficulty with everyday activities [34,35]. The Survey publications present data on disability in terms of the difficulties people experience in areas of daily life, as well as their impairments and long-term health conditions. The main environmental factor which informs SDAC’s definitions of disability is ‘assistance’. The Survey’s concepts of severe or profound core activity limitation relate to a person’s need for assistance with ‘core activities’, namely self-care, mobility and communication [35]. The operational definition of disability in SDAC is ‘any limitation, restriction or impairment which restricts everyday activities and has lasted, or is likely to last, for at least six months’ [36].

The SDAC methodology thus accords with that recommended by WHO authors and colleagues ([27], p. 1068): “… disability surveys should rely on a broad range of questions covering the whole range of the experience of functioning—bodily impairments, activity limitations, participation restrictions and environmental facilitators or barriers. Arguably, the final intention of health and health related interventions is to maximize functioning in a person’s lived environment and hence disability surveys are best focused on measuring the person’s functioning in interaction with his or her environment”.

Several other ABS social surveys include a ‘Short Disability Module’ of 16 questions that aims to identify people with disability and their limitations and restrictions in a way that aligns with SDAC [34]. A ‘Core Activity Need for Assistance’ module has been included in the Australian Census since 2006, to measure the number of people ‘needing help or assistance in one or more of the three core activity areas of self-care, mobility and communication, because of a long-term health condition (lasting six months or more), a disability (lasting six months or more), or old age’ [37]. It is designed to align conceptually with severe or profound core activity limitation in SDAC.

The use of common concepts and data standards relating to disability across population surveys and administrative data collections provides a basis for relating data from different sources. Australia’s disability services national minimum dataset, established in the early 2000s, included a ‘support needs’ data item based on the ICF activities and participation domains and the SDAC question on need for personal help or supervision with activities or participation in particular life areas [38]. The resulting data from the national disability services data collection could be related to SDAC data, which enabled some powerful analyses bringing together data on users of disability services with SDAC data on the target population to produce estimates and projections of met and unmet need for support [39]. Provision of disability services in Australia has now transitioned to the National Disability Insurance Scheme. Legislation governing eligibility for the Scheme requires that assessment tools used must “have reference to areas of activity and social and economic participation identified in the World Health Organisation International Classification of Functions, Disability and Health” ([40], 7.5(b)).

SDAC data are accessible to researchers external to the ABS, thus enabling wider use of the data. Data have been used, for example, to illuminate rights-related issues, such as the experience of disability discrimination, prevalence of disability among Indigenous Australians, and access to services by Australians of diverse cultures [41,42,43].

The CRPD sets out the rights of people with disability to access all services available to society as a whole. Creating a succinct disability ‘identifier’ for use in administrative data collections is a key challenge for public policy design and monitoring in any country [16,23,44]. Meeting this challenge involves the design and adoption of short question sets, ideally a single question, to identify people with disability consistently within service data systems, so that it is possible to monitor equity of access, participation and outcomes [45,46].

## 4. Data on Health and Health Service Use for People with Disability

According to SDAC 2018 data, more than 4 million Australians have disability, or around 18% of the population [2]. Lack of equitable and timely access to appropriate health care, especially preventive care and proactive management of health risks and chronic conditions, has been identified as a factor contributing to poor health outcomes for people with disability [4]. Submissions to Australia’s Royal Commission into Violence, Abuse, Neglect and Exploitation of People with Disability highlight barriers to equitable and timely access to quality health care for people with disability, particularly for those with cognitive disability and Aboriginal and Torres Strait Islander people with disability [47,48,49,50,51]. Barriers cited include inadequate staff training, expertise and capacity, limited access to skilled patient advocates, costs, accessibility of buildings and health care equipment (e.g., mammography machines), and discriminatory attitudes. There has long been recognition of the need to address health inequalities, and barriers to accessing appropriate health services, particularly for people with intellectual disability [52].

For people with disability, as for all people, health is a key enabler of participation across all domains of life, and health services, systems and policies are environmental factors that can play a critical role as facilitators of or barriers to participation. The Australian Health Performance Framework (AHPF) is used to assess the health of Australia’s population and performance of the health system [53,54,55]. It has four broad domains—Health status, Determinants of health, Health system, and Health system context. The principle of equity overarches these domains. To determine whether equity is being achieved, statistical data are required that enable indicators to be reported for particular population sub-groups, including people with disability.

### 4.1. What Do We Know about Health Conditions Experienced by People with Disability?

National population surveys provide data on health conditions experienced by people with and without disability. In SDAC, the survey modules used for disability identification ask respondents what is the main condition that causes each of the impairments or activity limitations they report. Respondents are then asked if they are receiving treatment for any long-term condition and whether they have any other long-term condition. Due to the structure of the survey questions in SDAC, there is an emphasis on health conditions that are associated with impairments and limitations. SDAC data have been used to report on rates of disability associated with particular health conditions, and on associations between health conditions and particular limitations and impairments [2,56,57,58]. For example, for people aged under 65 years in 2003, the health conditions with the highest associated rates of profound or severe core activity limitations were autism (82%), paralysis (79%), and speech-related conditions (67%); for people aged under 65 with severe or profound core activity limitations, the most common health conditions were back problems and arthritis, consistent with their high prevalence in the general population ([58], pp. 223–225).

The ABS National Health Survey also provides data on long-term health conditions, and identifies people with disability using the ABS short disability module. Table 1 presents data from the 2017–2018 National Health Survey for the 20 most common groups of current, long-term health conditions reported by all people aged 15–64 years, and compares prevalence for people with and without disability. ‘Disorders of ocular muscles, binocular movement accommodation & refraction’ were most prevalent, with similar rates for people with and without disability (65% and 62%, respectively). For all other condition groups listed, rates were higher for people with disability than for those without disability. For 11 condition groups, the rate for people with disability was more than twice that for people without disability. The rate for people with disability was more than three times that for people without disability for anxiety related problems, mood disorders, partial deafness and hearing loss, and other diseases of the ear.

National Health Survey data also reveal higher rates of multi-morbidity for people with disability compared to people without disability in 2017–2018. For people aged 15–24 years, the median number of current, long-term conditions reported was three for those with disability, compared to one for those without; for people aged 25–64 years, the median number of conditions was four for those with disability, compared to one for those without; and for people in the age groups 50–64 years and 65 years and over, the median number of conditions was six for those with disability, compared to three for those without.

These differences between people with and without disability in rates of long-term health conditions and multi-morbidity are not unexpected, given the relationship between health conditions and disability, discussed above. However, such data can shed light on the potential health-related needs of people with disability compared to those without disability. More detailed insights can be gained by breaking down the data, for example, by gender, disability group, disability severity, or area of residence (urban/non-urban). Such information is relevant for guiding health policy. For example, considering the higher rates of diabetes among people with disability, together with the prevalence of disability in the population, it is clear that diabetes prevention or management interventions must be accessible and appropriate for people with disability, otherwise they will not be effective for a substantial portion of the target population [11].

These data do not, however, provide insights into the extent to which higher rates of some conditions may be related to the right of equal access to healthcare not being met for people with disability, for instance, because of barriers to accessing primary and secondary prevention.

### 4.2. What Do We Know about Contact with Health Services by People with Disability?

Population surveys capture some information on contact with health services by people with disability. For instance, the National Health Survey (2017–18) provides data on the proportion of people who consulted different types of health professionals in the past 12 months. Compared to people without disability aged 18–64 years, a higher percentage of those with disability reported having consulted a GP (92%, compared with 83% of those without disability), a specialist (50%, compared with 29% of those without disability), and an allied health professional (37%, compared with 19% of those without disability) [59]. In the 2014 General Social Survey, a higher proportion of people with disability reported experiencing a barrier to accessing healthcare when needed (15%), compared to those without disability (3%); the proportion was higher still for people with severe or profound core activity limitation (24%) [59]. SDAC provides data on the proportion of people with disability who report unmet need for different types of health services, and who report cost as a barrier to accessing health services [2]; but these data are not collected for people without disability, so comparison is not possible.

While valuable, survey data on contact with health services, barriers, and unmet needs have a number of limitations. First, the available information is based on quite broad questions, so tends to lack specificity. Second, surveys capture a snapshot at certain points in time (e.g., every 3 years in the case of SDAC). Thirdly, the ability to conduct analyses focusing on particular subgroups of people with disability may be limited due to small sample size. Fourthly, the reliability of the information is dependent on the accuracy of respondents’ memory and perceptions about their interactions with health services.

In addition, survey sample frames for many ABS surveys do not cover people living in Very Remote Areas, in discrete Aboriginal and Torres Strait Islander communities, or in non-private dwellings. The ABS list of non-private dwellings includes boarding houses, hospitals, psychiatric hospitals or institutions, hostels for the disabled, nursing homes, accommodation for people who are homeless, prisons, and other welfare institutions (including group homes for people with disability) [37,60]. SDAC provides limited data on people who live in health establishments that provide long-term cared accommodation, but the questionnaire used for this survey component focuses on health conditions, core activities, use of aids, and assistance provided, and is completed by a staff member for each selected occupant; the data do not cover broader topics relating to contact with health services, participation across life domains, or social and economic outcomes.

Administrative data sources are an important complement to survey data for population heath research, as they can provide more detailed and comprehensive data on service users. Currently, in Australia, no national health services administrative datasets include disability identification. Therefore, it is not possible to produce policy-relevant data on contact with health services for people with disability and associated outcomes, or to make comparisons between people with and without disability to determine whether the AHPF objective of equity is being attained.

The National Hospitals Data Collection includes a number of databases that contain episode-level information on hospital care provided to admitted and non-admitted patients (https://www.aihw.gov.au/about-our-data/our-data-collections/national-hospitals; accessed on 5 November 2021). These databases are used for reporting on AHPF indicators, including rates of hospitalisation for injury and poisoning, hospitalisations involving an adverse event, selected potentially preventable hospitalisations, in-hospital falls, hospitalisation rates for selected procedures, waiting time to admission for selected elective surgery procedures, and emergency department presentations seen on time. None of these measures can be broken down by disability status, as there is no disability identifier in these data sources.

Consistent national data on primary health care (including GP visits) is currently not available. Data captured in patient electronic health records are not currently suitable for conducting reliable or representative analyses at regional, state or national levels, due to data quality, comparability and access issues [61,62]. Data from the now discontinued Bettering the Evaluation and Care of Health (BEACH) collection have been used to examine GP encounters for patients with intellectual disability and autism spectrum disorder, with analyses indicating that these patient groups differed from other patients in terms of demographic characteristics, reasons for encounter, consultation type, consultation length, problems managed, medications, treatments provided, and referrals made [63,64,65].

The Department of Health publishes statistics on services provided under Australia’s Medicare scheme (available to all Australians), for example, bulk-billing rates, average patient contributions, and broad service types (e.g., optometry, other allied health, diagnostic imaging). However, Medicare data cannot be broken down by patient disability status. (https://www1.health.gov.au/internet/main/publishing.nsf/Content/Medicare%20Statistics-1; accessed on 5 November 2021).

### 4.3. The Future Potential of Linked Administrative Data

Linked administrative datasets offer potential for filling some data gaps. The principle is that individual-level disability-identifying information captured in one dataset is linked with individual-level records in other datasets to enable analyses focusing on people with disability. This approach has been used in two Australian states to examine service use and related outcomes for people with intellectual disability [66,67,68,69,70], and to analyse mortality data for people with autism spectrum disorders [71]. For example, use of linked data to examine health service use in the last year of life for a matched cohort of people with and without intellectual disability in Western Australia found that people with intellectual disability attended emergency departments more frequently, were admitted to hospital less frequently, had longer hospital stays, and had increased odds of presentation, admission or death associated with potentially preventable conditions [72]. Similarly, a large New South Wales cohort study using linked data found intellectual disability to be associated with higher rates of emergency department presentations, psychiatric readmissions, premature mortality, and potentially avoidable deaths, and with longer length of stay in psychiatric units [66].

While data linkage has an important role to play in improving the evidence base, it must be understood that identification of people with disability in linked datasets will be limited by disability identifying information available in the constituent datasets. This is likely often to include identification based on receipt of disability-related services or income support payments. The majority of people with disability do not receive disability-related services or payments [2]. Thus, without a means of identifying people with disability more comprehensively in administrative data, information on equity of access to health and other services will remain incomplete.

## 5. Moving Forward: Some Key Considerations

We have outlined how the ICF model of disability is operationalised in data collections in Australia, to enable the disaggregation of population data by disability status, and we have provided an overview of available data on health and health service use for people with disability. It is clear that work is needed to address existing data gaps and limitations, to more fully understand the experiences of people with disability in relation to health and health services, to measure disability-related inequalities, and to monitor implementation of Article 25 of the CRPD. In this section, we raise some key considerations that must be kept front-of-mind as Australia moves forward with data development in this area.

There is a long history of research labelling, pathologising, and dehumanising people with disability [73,74]. The collection and use of statistical data is not a neutral exercise [75]. Just as the indigenous data sovereignty movement is a response to the statistical narrative of deficit and dysfunction that has framed the portrayal of indigenous peoples globally [76], so too are people with disability looking to take leadership over the way disability research is conducted, reported and utilised. Emerging out of the disability rights movement of the 1970s and 1980s, the discipline of disability studies builds on the logic of “nothing about us without us” to insist that only the active involvement of people with disability can protect research from the paternalism and medicalisation that has plagued its history. Co-produced research (often called inclusive research in the context of intellectual disability [77,78]) looks to guard against ableist bias and ensure that research is focused on social systems rather than individual deficits [79].

While the ICF itself is a product of the emerging disability rights movement, diligence is needed to ensure it is not used to label, classify and control people with disability. The ethical guidelines for the use of the ICF require respect for the autonomy of people with disability and their involvement in the collection and use of data ([21], Annex 6). These ideas can be deepened by including researchers with lived experience of disability and partnering with Disabled People’s Organisations in research design and the development of disability data and statistics, and in the use of data for informing public policy.

Among the ethical considerations that should guide the collection and reporting of statistical data on health and access to health services for people with disability is the need to ensure all cohorts of people are captured in data that will shape public policy. This requirement can be in tension with decisions to be made about research participation, such as the need to prevent coercion and judge the capacity for consent [80,81,82]. Contrary to common ableist presumptions (including among ethics committees) [83,84], it should be assumed that people with disability are capable of providing consent unless established otherwise [80]. Those gathering data have the responsibility to promote understanding about the purpose and intended use of the data ([21], Annex 6). This may require implementing supported decision-making practices, and tailoring approaches for gathering data that utilise mechanisms to facilitate understanding such as Easy Read [85,86].

Notwithstanding capacity to consent, many people with disability are reliant on gatekeepers, such as parents, support workers, and institutional managers, who may prevent or constrain their interaction with people gathering data [87]. Gatekeepers may be concerned about the impact of research upon ‘vulnerable’ people, and suspicious of its benefits. While suspicion of research and data collection is not without reason, overemphasis on protection discounts the harm of paternalism and undermines the rights of people with disability to participate in research that can inform improved disability policy and practices [86]. Both gatekeepers and people with disability themselves may be concerned that the information they provide could affect the way they are treated and their eligibility for supports [86]. As stated in the ICF ethical guidelines, data collected should enhance choices and control, support, and participation of people in the community, and not be employed to restrict people’s rights or entitlements ([21], Annex 6). Likewise, Article 31 of the CRPD requires the collection and use of statistical data to give effect to the rights set out in the Convention.

While confidentiality and privacy are central principles in the collection and management of data about individuals, some people with disability rely upon and trust their support workers, and depend upon their help in participating in research and providing data. Successful data collection is thus dependent upon fostering trust among complex stakeholder networks, utilising support to conduct research while collecting data using strict safeguards for identification, storage, and publication that protect participants from any negative consequences [80,86].

The challenges of collecting health data from and about people with disability are magnified in segregated institutional settings under the control of gatekeepers, yet monitoring of health is especially important in such settings. Particular mention should be made here of the criminal justice system. People with disability are known to be over-represented in the criminal justice system, with consequent negative health impacts [66,88,89,90], especially for those exposed to intersectional discrimination such as Aboriginal and Torres Strait Islander people with disability [91]. There is also evidence that justice systems often fail to identify offenders with intellectual disability [89,90]. Thus, to ensure rights to health and health services are upheld, strategies are needed to ensure the full representation of people with disability within these settings.

Producing statistics is not just a technical endeavour. We have already identified the centrality of co-producing health data with people with disability, and this rationale extends to including the insight of people with disability and other intersectional health disadvantages, such as racial, sexual, gendered, and aged. For example, Aboriginal and Torres Strait Islander people with disability experience multilayered disadvantage from racism and ableism, and collecting and publishing health statistics on this cohort of people is thus vital and complex [92]. It involves recognition of cultural, social, political and ethical dimensions of research with First Peoples with disability, especially their right to self-determination, and culturally safe and inclusive research practices [93,94].

## 6. Conclusions

In Australia, we are fortunate to have established population data sources with disability identifiers that are conceptually aligned with the ICF. This is a result of sustained resourcing by government, the work of statistical agencies (particularly ABS and AIHW), and active input from people with disability and other stakeholders. Nonetheless, important data gaps and limitations remain, hampering Australia’s ability to develop effective policy responses to uphold the rights of people with disability, especially in relation to health and access to health services.

Moving forward to address these data gaps and limitations, the challenge is to develop an array of sources, with relatable data on disability. Clear articulation of the purpose for which data are to be used is crucial to guide any data development work, but particularly in relation to identifying people with disability, and deciding where and how to place ‘cut points’ along the continuum of functioning to define different groups. The corollary of this is that the purpose for which a particular data source has been designed must be understood and taken into account when considering secondary use of the data, including in the context of data linkage.

Disability data development should adhere to well-established principles, such as using data standards to promote quality and consistency, the importance of consultation, collaboration and field testing, being aware of the limitations of the data, and weighing the costs of data collection to all concerned against the value gained [17,95,96].

Building an effective evidence base to inform better policy and practice requires that data from different sources are relatable and have sound conceptual underpinnings; ICF remains the relevant standard framework in relation to disability. However, developing data is never merely a technical endeavour: there must always be consideration of the cultural, social, political and ethical dimensions, and the implications for those to whom the data relate. Crucially, people with disability and their representative organisations must be key players in the development of disability data and statistics, and in their use.

Finally, good data rely on ongoing, active input from all parties who have an interest in the data. We encourage disability advocates, researchers, and policy makers to use the valuable disability data sources available in Australia, with an awareness of the concepts underpinning these data, and to engage in ongoing discussions and efforts to improve our national disability data resources into the future.

## Figures and Tables

**Table 1:** Prevalence of the 20 most commonly reported ^1^ current, long-term health conditions, by disability status, for people aged 15–64 years (National Health Survey 2017–2018).

Health Condition Group	Disability (95% CI)	No Disability ^2^ (95% CI)	Rate Ratio ^3^ (95% CI)
1. Disorders of ocular muscles binocular movement accommodation & refraction	65% (63, 67)	62% (61, 63)	1.0 (1.0, 1.1)
2. Other diseases of respiratory system	32% (30, 34)	25% (24, 26)	1.3 (1.2, 1.4)
3. Dorsopathies	37% (35, 39)	13% (12, 14)	2.9 (2.6, 3.2)
4. Symptoms, signs and conditions not elsewhere classified (NEC)	24% (22, 25)	12% (11, 13)	1.9 (1.8, 2.1)
5. Anxiety related problems	29% (27, 31)	9% (8, 10)	3.4 (3.0, 3.8)
6. Mood (affective) disorders	29% (27, 31)	7% (6, 8)	4.2 (3.6, 4.8)
7. Arthropathies	28% (26, 29)	10% (9, 11)	2.7 (2.5, 3.0)
8. Chronic lower respiratory diseases	20% (18, 22)	9% (9, 10)	2.1 (1.8, 2.4)
9. Episodic & paroxysmal disorders	18% (16, 19)	6% (5, 7)	3.0 (2.6, 3.4)
10. Hypertensive disease	13% (12, 15)	8% (7, 9)	1.6 (1.4, 1.8)
11. Partial deafness & hearing loss (NEC)	20% (19, 21)	3% (3, 4)	5.7 (4.7, 6.6)
12. Other endocrine, nutritional & metabolic diseases	10% (9, 12)	5% (5, 6)	2.0 (1.6, 2.3)
13. Other diseases of the ear	10% (9, 12)	2% (2, 3)	4.1 (3.1, 5.1)
14. Diseases of the skin and subcutaneous tissue	6% (5, 7)	4% (3, 4)	1.6 (1.2, 2.0)
15. Diabetes mellitus	6% (5, 7)	4% (3, 4)	1.7 (1.4, 2.1)
16. Disorders of thyroid gland	6% (5, 7)	4% (3, 4)	1.6 (1.2, 1.9)
17. Diseases of genito-urinary system	6% (5, 7)	3% (2, 3)	2.3 (1.7, 2.8)
18. Other diseases of eye & adnexa	4% (3, 5)	3% (2, 3)	1.4 (1.1, 1.8)
19. Diseases of the oesophagus, stomach & duodenum	5% (4, 6)	2% (1, 2)	2.6 (1.9, 3.3)
20. Diseases of blood and blood forming organs	4% (3, 4)	2% (1, 2)	2.1 (1.6, 2.7)

^1^ Based on health conditions reported by all people aged 15–64 years. ^2^ Prevalence rates for people with ‘no disability’ are age-standardised to the age structure of the population with disability; these rates should be interpreted as the hypothetical rates that would have been observed if the population without disability had the same age structure as the population with disability. ^3^ Rate ratio is calculated as: (% for people with disability)/(% for people without disability). *Source*: Australian Bureau of Statistics (2019) Microdata: National Health Survey, 2017–2018 (DataLab), accessed July 2021.

## Data Availability

ABS survey data cited in this paper are available to researchers for analysis, see https://www.abs.gov.au/websitedbs/d3310114.nsf/home/microdata+entry+page (accessed on 5 November 2021).

## References

[1] United Nations Convention on the Rights of Persons with Disabilities. www.un.org/development/desa/disabilities/convention-on-the-rights-of-persons-with-disabilities.html.

[2] Australian Institute of Health and Welfare (2020). People with Disability in Australia 2020.

[3] Australian Institute of Health and Welfare (2020). Mortality Patterns Among People Using Disability Support Services: 1 July 2013 to 30 June 2018.

[4] Salomon C., Trollor J. (2019). A Scoping Review of Causes and Contributors to Deaths of People with Disability in Australia (2013–2019). Exhibit 4-059-CTD.7200.0001.0060 3DN, UNSW. Report for the Royal Commission into Violence, Abuse, Neglect and Exploitation of People with Disability.

[5] Trollor J., Small J. (2019). Health Inequality and People with Intellectual Disability—Research Summary.

[6] United Nations (2019). Disability and Development Report: Realizing the Sustainable Development Goals by, for and with Persons with Disabilities, 2018.

[7] Krahn G.L., Walker D.K., Correa-De-Araujo R. (2015). Persons with disabilities as an unrecognized health disparity population. Am. J. Public Health.

[8] United Nations Committee on the Rights of Persons with Disabilities (2019). Concluding Observations on the Combined Second and Third Periodic Reports of Australia.

[9] Davy L., Fisher K., Wehbe A., Purcal C., Robinson S., Kayess R., Santos D. (2018). Review of Implementation of the National Disability Strategy 2010–2020. Final Report.

[10] Civil Society CRPD Shadow Report Working Group Disability Rights Now 2019. Australian Civil Society Shadow Report to the United Nations Committee on the Rights of Persons with Disabilities: UN CRPD Review 2019. https://pwd.org.au/our-work/policy-areas/human-rights-campaigns/united-nations-convention-on-the-rights-of-persons-with-disabilities/crpd-civil-society-shadow-report/.

[11] Krahn G. (2021). Where is disability in global public health?. Oxford Research Encyclopedia of Global Public Health.

[12] Moon L., Gourley M., Goss J., On M.L., Laws P., Reynolds A., Juckes R. (2020). History and development of national burden of disease assessment in Australia. Arch. Public Health.

[13] Vos T., Lim S.S., Abbafati C., Abbas K.M., Abbasi M., Abbasifard M., Abbasi-Kangevari M., Abbastabar H., Abd-Allah F., Abdelalim A. (2020). Global burden of 369 diseases and injuries in 204 countries and territories, 1990–2019: A systematic analysis for the Global Burden of Disease Study 2019. Lancet.

[14] Hancock A. Best Practice Guidelines for Developing International Statistical Classifications. United Nations Department of Economic and Social Affairs Statistics Division. Expert Group Meeting on International Statistical Classifications. New York, 13–15 May 2013. https://unstats.un.org/unsd/classifications/bestpractices/Best_practice_Nov_2013.pdf.

[15] World Health Organization (2016). International Statistical Classification of Diseases and Related Health Problems.

[16] World Health Organization, World Bank (2011). World Report on Disability.

[17] Madden R., Madden R., Australian Institute of Health and Welfare (2019). Disability services and statistics: Past, present and future. Australia’s Welfare 2019 Data Insights.

[18] Centre for Disability Research and Policy (2017). Audit of Disability Research in Australia Update Report 2017.

[19] Ustun T.B., Chatterji S., Bickenbach J., Kostanjsek N., Schneider M. (2003). The International Classification of Functioning, Disability and Health: A new tool for understanding disability and health. Disabil Rehabil.

[20] Schneidert M., Hurst R., Miller J., Üstün B. (2003). The role of environment in the International Classification of Functioning, Disability and Health (ICF). Disabil. Rehabil..

[21] World Health Organization (2001). International Classification of Functioning, Disability and Health.

[22] Bickenbach J.E. (2009). Disability, culture and the UN convention. Disabil. Rehabil..

[23] Bickenbach J.E. (2011). Monitoring the United Nation’s Convention on the Rights of Persons with Disabilities: Data and the International Classification of Functioning, Disability and Health. BMC Public Health.

[24] Madden R.H., Bundy A. (2018). The ICF has made a difference to functioning and disability measurement and statistics. Disabil. Rehabil..

[25] United Nations Fundamental Principles of Official Statistics (A/RES/68/261 from January 2014). https://unstats.un.org/fpos/.

[26] Madden R., Sykes C., Üstün B. (2007). World Health Organization Family of International Classifications: Definition, Scope and Purpose.

[27] Kostanjsek N., Good A., Madden R.H., Üstün T.B., Chatterji S., Mathers C.D., Officer A. (2013). Counting disability: Global and national estimation. Disabil. Rehabil..

[28] World Health Organization How to Use the ICF: A Practical Manual for Using the International Classification of Functioning, Disability and Health (ICF). Exposure Draft for Comment. http://www.who.int/entity/classifications/drafticfpracticalmanual2.pdf?ua=1.

[29] World Health Organization (2015). WHO Global Disability Action Plan 2014–2021. Better Health for All People with Disability.

[30] Commonwealth of Australia (2011). 2010–2020 National Disability Strategy. An initiative of the Council of Australian Governments.

[31] National Disability Insurance Scheme Act 2013 (Cth). https://www.legislation.gov.au/Details/C2020C00392.

[32] Madden R., Fortune N., Cheeseman D., Mpofu E., Bundy A. (2013). Fundamental questions before recording or measuring functioning and disability. Disabil. Rehabil..

[33] World Health Organization (2017). Model Disability Survey (MDS): Survey Manual.

[34] Australian Bureau of Statistics (2018). 4431.0.55.002—ABS Sources of Disability Information, 2012–2016.

[35] Australian Bureau of Statistics (2019). Disability, Ageing and Carers, Australia: Summary of Findings Methodology, 2018.

[36] Australian Bureau of Statistics (2019). 4430.0—Disability, Ageing and Carers, Australia: Summary of Findings, 2018.

[37] Australian Bureau of Statistics (2016). 2901.0—Census of Population and Housing: Census Dictionary, 2016.

[38] Anderson P., Madden R. (2011). Design and quality of ICF-compatible data items for national disability support services. Disabil. Rehabil..

[39] Australian Institute of Health and Welfare (2007). Current and Future Demand for Specialist Disability Services.

[40] Australian Government (2016). National Disability Insurance Scheme (Becoming a Participant) Rules. https://www.legislation.gov.au/Details/F2018C00165.

[41] Coleman C., Man N.W.Y., Gilroy J., Madden R. (2018). Aboriginal and Torres Strait Islander disability prevalence: Making sense of multiple estimates and definitions. Aust. N. Z. J. Public Health.

[42] Temple J.B., Kelaher M., Williams R. (2018). Discrimination and avoidance due to disability in Australia: Evidence from a National Cross Sectional Survey. BMC Public Health.

[43] Zhou Q. (2016). Accessing disability services by people from culturally and linguistically diverse backgrounds in Australia. Disabil. Rehabil..

[44] Verbrugge L.M., Jette A.M. (1994). The disablement process. Soc. Sci. Med..

[45] Madden R., Choi C., Sykes C. (2003). The ICF as a framework for national data: The introduction of ICF into Australian data dictionaries. Disabil. Rehabil..

[46] Madden R.H., Lukersmith S., Zhou Q., Glasgow M., Johnston S. (2020). Disability-related questions for administrative datasets. Int. J. Environ. Res. Public Health.

[47] Lowitja Institute Submission to the Royal Commission into Violence, Abuse, Neglect and Exploitation of People with Disability. Issues Paper: Health Care for People with Cognitive Disability. May 2020. https://disability.royalcommission.gov.au/publications/iss00100228.

[48] New South Wales Ageing and Disability Commission Submission to the Royal Commission into Violence, Abuse, Neglect and Exploitation of People with Disability. Issues Paper: Health Care for People with Cognitive Disability. February 2020. https://disability.royalcommission.gov.au/publications/iss00100038.

[49] Royal Australian College of General Practitioners RACGP Submission to the Royal Commission into Violence, Abuse, Neglect and Exploitation of People with Disability. Issues Paper: Healthcare for People with Cognitive Disability. April 2020. https://www.racgp.org.au/getmedia/06c7a743-30aa-42c5-b53b-6a4b629ab222/RACGP-submission-Healthcare-for-people-with-cognitive-disability.pdf.aspx.

[50] Queensland Aboriginal and Islander Health Council QAIHC Submission to the Royal Commission into Violence, Abuse, Neglect and Exploitation of People with Disability. April 2020. https://www.qaihc.com.au/media/37598/200406-submission-health-care-for-people-with-cognitive-disability-v11-final-20200407.pdf.

[51] Avery S. Royal Commission into Violence, Abuse, Neglect and Exploitation of People with Disability. Statement of Dr Scott Avery. 14 February 2020. https://disability.royalcommission.gov.au/publications/exhibit-4-18-stat006500010001-statement-dr-scott-avery.

[52] Beange H., Lennox N., Parmenter T.R. (1999). Health targets for people with an intellectual disability. J. Intellect. Dev. Disabil..

[53] Australian Institute of Health and Welfare (2018). Australia’s Health 2018.

[54] National Health Information and Performance Principal Committee (2017). The Australian Health Performance Framework.

[55] Australian Institute of Health and Welfare Australian Health Performance Framework. https://www.aihw.gov.au/reports-data/australias-health-performance.

[56] Australian Institute of Health and Welfare (2018). Chronic Conditions and Disability 2015.

[57] Australian Institute of Health and Welfare (2004). Disability and Its Relationship to Health Conditions and other Factors.

[58] Australian Institute of Health and Welfare (2005). Australia’s Welfare 2005.

[59] Fortune N., Badland H., Stancliffe R.J., Emerson E., Llewellyn G. (2021). Disability and Wellbeing Monitoring Framework: Baseline Indicator Data for Australians Aged 18–64 Years.

[60] Fortune N., Badland H., Clifton S., Emerson E., Rachele J., Stancliffe R.J., Zhou Q., Llewellyn G. (2020). The Disability and Wellbeing Monitoring Framework: Data, data gaps, and policy implications. Aust. N. Z. J. Public Health.

[61] Canaway R., Boyle D.I., Manski-Nankervis J.A.E., Bell J., Hocking J.S., Clarke K., Clark M., Gunn J.M., Emery J.D. (2019). Gathering data for decisions: Best practice use of primary care electronic records for research. Med. J. Aust..

[62] Bailie R., Bailie J., Chakraborty A., Swift K. (2015). Consistency of denominator data in electronic health records in Australian primary healthcare services: Enhancing data quality. Aust. J. Prim. Health.

[63] Foley K.-R., Pollack A.J., Britt H.C., Lennox N.G., Trollor J.N. (2018). General practice encounters for young patients with autism spectrum disorder in Australia. Autism.

[64] Weise J., Pollack A., Britt H., Trollor J. (2017). Primary health care for people with an intellectual disability: An exploration of consultations, problems identified, and their management in Australia. J. Intellect. Disabil. Res..

[65] Weise J., Pollack A., Britt H., Trollor J.N. (2016). Primary health care for people with an intellectual disability: An exploration of demographic characteristics and reasons for encounters from the BEACH programme. J. Intellect. Disabil. Res..

[66] Reppermund S., Heintze T., Srasuebkul P., Reeve R., Dean K., Smith M., Emerson E., Snoyman P., Baldry E., Dowse L. (2019). Health and wellbeing of people with intellectual disability in New South Wales, Australia: A data linkage cohort. BMJ Open.

[67] Reppermund S., Srasuebkul P., Dean K., Trollor J.N. (2020). Factors associated with death in people with intellectual disability. J. Appl. Res. Intellect. Disabil..

[68] Reppermund S., Srasuebkul P., Heintze T., Reeve R., Dean K., Emerson E., Coyne D., Snoyman P., Baldry E., Dowse L. (2017). Cohort profile: A data linkage cohort to examine health service profiles of people with intellectual disability in New South Wales, Australia. BMJ Open.

[69] Florio T., Trollor J. (2015). Mortality among a cohort of persons with an intellectual disability in New South Wales, Australia. J. Appl. Res. Intellect. Disabil..

[70] Balogh R., Leonard H., Bourke J., Brameld K., Downs J., Hansen M., Glasson E., Lin E., Lloyd M., Lunsky Y. (2019). Data linkage: Canadian and Australian perspectives on a valuable methodology for intellectual and developmental disability research. IDD.

[71] Hwang Y.I., Srasuebkul P., Foley K.R., Arnold S., Trollor J.N. (2019). Mortality and cause of death of Australians on the autism spectrum. Autism Res..

[72] Brameld K., Spilsbury K., Rosenwax L., Leonard H., Semmens J. (2018). Use of health services in the last year of life and cause of death in people with intellectual disability: A retrospective matched cohort study. BMJ Open.

[73] Linton S. (1998). Disability Studies/Not Disability Studies. Disabil. Soc..

[74] Bruce C. (2020). Unsettling ableism in research traditions: Toward establishing blind methodologies. Social Research and Disability.

[75] Clifton S., Fortune N., Llewellyn G., Stancliffe R.J., Williamson P. (2020). Lived expertise and the development of a framework for tracking the social determinants, health, and wellbeing of Australians with disability. Scand. J. Disabil. Res..

[76] Judith W., Gillian K., Neil D., Katie W. (2018). Indigenous data sovereignty in higher education: Towards a decolonised data quality framework. Aust. Univ. Rev..

[77] Nind M. (2014). What Is Inclusive Research?.

[78] Bigby C., Frawley P., Ramcharan P. (2014). Conceptualizing Inclusive Research with People with Intellectual Disability. J. Appl. Res. Intellect. Disabil..

[79] Roennfeldt H., Byrne L. (2020). Lived experience researchers: Opportunities and challenges in mental health. Social Research and Disability.

[80] Dalton A.J., McVilly K.R. (2004). Ethics Guidelines for International, Multicenter Research Involving People with Intellectual Disabilities1,2,3,4. J. Policy Pract. Intellect. Disabil..

[81] McDonald K.E., Kidney C.A. (2012). What is right?. Ethics in intellectual disabilities research. J. Policy Pract. Intellect. Disabil..

[82] Taua C., Neville C., Hepworth J. (2014). Research participation by people with intellectual disability and mental health issues: An examination of the processes of consent. Int. J. Ment. Health Nurs..

[83] McDonald K.E., Keys C.B., Henry D.B. (2008). Gatekeepers of science: Attitudes toward the research participation of adults with intellectual disability. Am. J. Ment. Retard..

[84] Mello A.G.D. (2016). Deficiência, incapacidade e vulnerabilidade: Do capacitismo ou a preeminência capacitista e biomédica do Comitê de Ética em Pesquisa da UFSC. Ciência Saúde Coletiva.

[85] Sutherland R.J., Isherwood T. (2016). The Evidence for easy-read for people with intellectual disabilities: A Systematic Literature Review. J. Policy Pract. Intellect. Disabil..

[86] McDonald K.E., Conroy N.E., Olick R.S., Panel T.P.E.E. (2017). What’s the harm?. Harms in research with adults with intellectual disability. Am. J. Intellect. Dev. Disabil..

[87] Williams P. (2020). ‘It all sounds very interesting, but we’re just too busy!’: Exploring why ‘gatekeepers’ decline access to potential research participants with learning disabilities. Eur. J. Spec. Needs Educ..

[88] Baldry E., Briggs D.B., Goldson B., Russell S. (2018). ‘Cruel and unusual punishment’: An inter-jurisdictional study of the criminalisation of young people with complex support needs. J. Youth Stud..

[89] Ellem K., Wilson J., Chui W.H., Knox M. (2008). Ethical challenges of life story research with ex-prisoners with intellectual disability. Disabil. Soc..

[90] Vanny K., Levy M., Hayes S. (2008). People with an intellectual disability in the Australian criminal justice system. Psychiatry Psychol. Law.

[91] McCausland R., Baldry E. (2017). ‘I feel like I failed him by ringing the police’: Criminalising disability in Australia. Punishm. Soc..

[92] Temple J.B., Wong H., Ferdinand A., Avery S., Paradies Y., Kelaher M. (2020). Exposure to interpersonal racism and avoidance behaviours reported by Aboriginal and Torres Strait Islander people with a disability. Aust. J. Soc. Issues.

[93] Avery S. (2018). Culture is Inclusion: A narrative of Aboriginal and Torres Strait Islander People with Disability.

[94] Walsh C., Puszka S. (2021). Aboriginal and Torres Strait Islander Voices in Disability Support Services: A Collation of Systematic Reviews.

[95] Australian Institute of Health and Welfare (2007). A Guide to Data Development.

[96] World Health Organization, United Nations ESCAP (2008). Training Manual on Disability Statistics.

